# Transcriptional and Functional Plasticity Induced by Chronic Insulin Exposure in a Mast Cell-Like Basophilic Leukemia Cell Model

**DOI:** 10.4172/2476-1966.1000135

**Published:** 2017-12-11

**Authors:** Chad Jansen, Mark Speck, William E Greineisen, Kristina Maaetoft-Udsen, Edward Cordasco, Lori MN Shimoda, Alexander J Stokes, Helen Turner

**Affiliations:** 1 Laboratory of Immunology and Signal Transduction, Chaminade University, Honolulu, Hawai’i, USA; 2 Undergraduate Program in Biochemistry, Chaminade University, Honolulu, Hawai’i, USA; 3 Laboratory of Experimental Medicine, Department of Cell and Molecular Biology, John A. Burns School of Medicine, University of Hawai’i, Honolulu, Hawai’i, USA

**Keywords:** Secretory granules, Lipid bodies, Mast cells, Plasticity, Hyperinsulinemia, Chronic insulin exposure, Immune system

## Abstract

**Objective:**

Secretory granules (SG) and lipid bodies (LB) are the primary organelles that mediate functional responses in mast cells. SG contains histamine and matrix-active proteases, while LB are reservoirs of arachidonic acid and its metabolites, precursors for rapid synthesis of eicosanoids such as LTC_4_. Both of these compartments can be dynamically or ontologically regulated, with metabolic and immunological stimuli altering lipid body content and granule numbers responding to contextual signals from tissue. We previously described that chronic *in vitro* or *in vivo* hyperinsulinemia expands the LB compartment with a concomitant loss of SG capacity, suggesting that this ratio is dynamically regulated. The objective of the current study is to determine if chronic insulin exposure initiates a transcriptional program that biases model mast cells towards a lipogenic state with accompanying loss of secretory granule biogenesis.

**Methods:**

We used a basophilic leukemic cell line with mucosal mast cell-like features as a model system. We tested the hypothesis that chronic insulin exposure initiates a transcriptional program that biases these model mast cells towards a lipogenic state with accompanying loss of secretory granule biogenesis. Transcriptional arrays were used to map gene expression patterns. Biochemical, immunocytochemical and mediator release assays were used to evaluate organelle numbers and functional responses.

**Results:**

In a mucosal mast cell model, the rat basophilic leukemia line RBL2H3, mast cell granularity and SG numbers are inversely correlated with LB numbers. Chronic insulin exposure appears to modulate gene networks involved in both lipid body biogenesis and secretory granule formation. Western blot analysis confirms upregulation of protein levels for LB proteins, and decreases in proteins that are markers for SG cargo.

**Conclusions:**

The levels of insulin in the extracellular milieu may modify the phenotype of mast cell-like cells *in vitro*.

## Introduction

A number of immune system cell types bear the insulin receptor, providing for the coupling of endocrine and dietary status to the intensity and duration of immune responses [1–3]. Insulin fluctuates daily in the human body altering the extracellular milieu in the circulation and certain tissues such as gut, brain and adipose [4]. At the population level, plasma insulin levels are distributed between two poles of hypo (Type I and II diabetes mellitus) and hyper (insulin resistance and the Metabolic Syndrome, MS) insulinemia. Hyperinsulinemia is of particular concern since it is a persistent state, lasting decades to years during the development of the Metabolic Syndrome. With Metabolic Syndrome at epidemic levels, it is timely to examine the impact of a chronically elevated insulin milieu upon the functionality of immunocytes.

The levels of insulin in the extracellular milieu are of relevance to mast cells and basophils, and in turn, the functionality of these cells is of relevance to outcomes in metabolic disorders [5–7]. Mast cells and basophils bear the insulin receptor, and are regulated by insulin levels. Examples of this regulation include direct evidence for insulin promotion of proliferation in immature bone marrow derived mast cells (BMMC) [8], and at the whole organism level a linkage between insulin and mast cell pro-inflammatory potential is indicated by a number of studies showing that chemically or surgically-pancreatectomized mice are defective in anaphylactic responses despite basically normal mast cell numbers [9,10]. Mast cells appear to contribute to outcomes in aspects of the metabolic syndrome, [11,12] especially in adipose depots [13]. Shi et al. noted reduced adiposity and reduced MS symptoms in mast cell deficient mice fed a high fat diet, compared to wild type animals, and in a later study documented that IgE and mast cell proteases are associated with impaired glucose tolerance [14].

Mast cells and basophils that are chronically exposed to elevated insulin, either *in vitro* or *in vivo* as a consequence of high fat diet induced obesity, display altered morphology and functional responses [15]. The insulin-induced altered phenotype is characterized by the accumulation of large numbers of lipid bodies, achieving steatotic levels in the cytoplasm. Lipidomic analysis shows that these lipid bodies are distinct from the neutral lipid storage droplets induced by insulin/ caloric overload in adipocytes and hepatocytes, with the mast cell lipid body content being enriched in fatty acids including arachidonate, omega poly-unsaturated fatty acids and arachidonic acid precursors and metabolites. This new pool of precursors for the synthesis of bioactive lipids such as leukotriene C_4_ translates to enhanced synthesis and release of LTC_4_ and other bioactive lipids in response to antigenic stimuli [6]. This gain of function in the bioactive lipid arm of the mast cell functional responses is accompanied by an intriguing loss of function; insulin exposed mast cells and basophils exhibit decreased granularity and secretory granule number, with concomitantly suppressed histamine release in response to antigen. Thus mast cells and basophils exhibit a type of functional plasticity induced by insulin. This insulin-induced altered mast cell phenotype is the subject of further study in this paper.

In this study, we analyzed the transcriptome of normal and chronically insulin-exposed cells in a basophilic leukemia line that recapitulates many functional features of mast cells. We tested the hypothesis that within this transcriptional program there is a signature associated with functional plasticity, particularly gains of function in lipid body biogenesis and losses of function in secretory granule biogenesis. We examined significant differentially expressed genes and the biological processes associated with hyperinsulinemic treatments. Gene ontology (GO) enrichment using Kolmogorov-Smirnov tests revealed differential regulation of lipid body biogenesis, lipid synthesis pathways and differential regulation of genes involved in secretory granule pathways. Together with prior studies, these data suggest that insulin alters model mast cell phenotype when these cells are exposed to high insulin levels chronically in culture.

## Materials and Methods

### Cell culture

RBL2H3 [16] were grown at 37°C, 5% CO_2_, in 95% humidity in Dulbecco’s Modification of Eagle Medium (Mediatech Inc., Herndon, VA) with 10% heat-inactivated Fetal Bovine Serum (Mediatech) and 2 mM Glutamine. 3T3-L1 were grown at 37°C, 5% CO_2_, in 95% humidity in Dulbecco’s Modification of Eagle Medium (Mediatech Inc., Herndon, VA) with 10% heat-inactivated Bovine Calf Serum (Hyclone), 2 mM Glutamine and 1 mM Sodium pyruvate.

### Chemicals and reagents

General chemicals were from VWR (West Chester, PA). Phorbol-12 myristate 13-acetate (PMA) and ionomycin were from EMD Millipore (Gibbstown, NJ). Anti-mast cell tryptase and anti-perilipin A/B were from AbCam (Cambridge, MA), Alexa-conjugated secondary antibodies were from Molecular Probes (Eugene, OR) and HRP conjugated secondary antibodies were from Amersham GE Healthcare (Piscataway, NJ). Porcine glucagon was from Sigma (St. Louis, MO) and recombinant rat TNF alpha was from R and D Systems (Minneapolis, MN).

### Cell stimulation

FcεRI stimulation used 0.1 µg/ml IgE anti-DNP (16 h/37°C) followed by three washes and the addition of 250 ng/ml KLH-DNP for indicated times.

### Insulin exposure and lipogenesis

Optimal lipogenesis is routinely achieved *in vitro* through addition of insulin, in combination with an inhibitor of autocrine TNF alpha production and stabilization of cAMP levels. Insulin drives lipogenesis while the corticosteroid dexamethasone opposes constitutive lipolysis through inhibiting TNF alpha production (a lipolytic cytokine) and downregulation of Hormone Sensitive Lipase (HSL) levels [17–20]. Chronic insulin exposure was a 6 day protocol. Insulin was used in at 2 µg/ml in combination with 250 nM dexamethasone and 100 nM IBMX (day 1) and then the remaining 5 days of the stimulation were insulin (2 µg/ml) alone [17–20]. Glucagon and TNF alpha were used at 10 µM and 20 ng/ml respectively. Lipogenesis was confirmed by LipidTOX (Invitrogen, Temecula, CA) staining as described [6,21].

### Flow cytometry

Cells were fixed in 0.4% paraformaldehyde (45 min, RT) and resuspended at 1 × 10^6^ cells/ml in FACS buffer (HBSS, 0.5% BSA, 0.05% NaN_3_). For lipid body analysis, cells were stained with Nile Red (0.1 µg/ml, 30 min RT). Fluorescence was assessed on a FACSAria Flow Cytometer (BD Biosciences, Franklin Lakes, NJ) at the John A. Burns School of Medicine Flow Cytometry Facility, University of Hawaii. Flow cytometry data were analysed in FlowJo version 9.02.

### Toluidine blue staining

Cells were fixed in 0.4% paraformaldehyde (1 h, RT) and blocked with 0.75% (w/v) fish skin gelatin (FSG) (1 h, RT). Cells were permeabilized with 0.4% Triton X-100 (4 min, RT). Cells were then stained with DAPI and Lipitox green (45 min, RT). During last 30 min of staining, toluidine blue was added for secretory granule staining. Cells were then washed three times with 1X PBS and mounted with Clear-Mount (Electron Microscopy Services) onto coverslips and analysed under epifluorescence or confocal microscopy.

### Microarray processing

RNA was extracted from 6-day Insulin and control RBL2H3 mast cells using the RNeasy Plus Mini Kit (Qiagen, Hilden, Germany), verified using an Agilent 2100 Bioanalyzer (Agilent, Santa Clara, CA), and total concentration determined using NanoVue (Healthcare Biosciences, Uppsala, Sweden). RNA was amplified and labeled using the Agilent Quick amp aRNA labeling kit (Agilent, Santa Clara, CA) according to the manufacturer’s instructions. After labeling, antisense RNA (aRNA) was fragmented using Agilent Gene Expression Hybridization Kit (Agilent Technologies, Palo Alto, CA). The fragmented aRNA was hybridized to the 4×44K Whole Rat Genome Microarray (Agilent Technologies, Palo Alto, CA) comprising 45,000 probe sets representing over 41,000 rat genes. The arrays were washed and scanned according to the manufacturer’s instructions at the John A. Burns School of Medicine the Genomics Core Facility, University of Hawaii (Honolulu, HI).

### Microarray analysis

The microarray data was analyzed using R and Bioconductor [22]. Raw probe intensities were normalized using affy and expression index calculations were performed using fRMA [23,24]. For statistical testing, a two-way ANOVA was applied and the false discovery rate (FDR) was estimated using a Monte Carlo approach. The statistical significance was set at an FDR of 0. Gene Set Enrichment (GSEA) was performed applying the method described by Kim and Volsky [25] in R using the PGSEA package [26]. For this analysis only genes with an absolute fold change greater than 2 were included. Gene network graphs were generated in Cytoscape v3.1.0 [27]. Any confirmatory real-time qPCR analysis was performed as follows: DNA was synthesized from 600 ng total RNA using the High-Capacity cDNA transcriptase kit (Applied Biosystems, Foster City, CA, USA). TaqMan Gene Expression Assays were used (Applied Biosystems) for IL-6 (Assay ID: Rn01410330_m1) and β-actin (Assay ID: Rn00667869_m1). The amplifications were carried out in 10 µl containing 1× Taqman Fast Universal Master Mix, 1× Taqman Gene Expression Assay, and purified target cDNA. The cycling parameters were 30 s at 94°C, 40 cycles at 94°C for 3 s, and 60°C for 30 s using the StepOnePlus (Applied Biosystems). Amplifications were performed in triplicate. Signals were normalized to expression of β-actin. Analysis used the method of Pfaffl [28].

### Cell lysis and western blots

Mast cells were lysed (ice/30 min) in 350 µL of lysis buffer (50 mM Hepes pH 7.4, 250 mM NaCl, 20 mM NaF, 10 mM iodoacetamide, 0.5% (w/v) Triton X100, 1 mM PMSF (phenylmethylsulfonylfluoride), 500 µg/ml aprotinin, 1.0 mg/ml leupeptin and 2.0 mg/ml chymostatin). Lysates were clarified (17,000 g, 20 min) and acetone precipitated (1.4 volumes acetone, 1 h/−20°C, followed by 10,000 g, 5 min). Protein was resolved by 10% reducing SDS-PAGE in a modified Laemmli buffer and electro-transferred to PVDF in 192 mM glycine, 25 mM Tris (pH 8.8). Membranes were blocked (5% non-fat milk in PBS, 1 h, RT) and probed (primary antibodies in PBS/0.05% Tween-20/0.05% NaN_3_, 16 h/4°C). Developing antibodies comprised anti-rabbit or anti-mouse IgGs conjugated to horseradish peroxidase (Amersham) at 0.1 µg/ml in PBS/0.05% Tween-20 (45 min/RT). Signal was visualized using ECL (Amersham) and Kodak BioMax film. Films were scanned at >600 dpi and quantification was performed using Image J (NIH).

### ORO staining

Cells (50,000 per cm^2^) coverslips were fixed on coverslips (0.4% (w/v) paraformaldehyde 1 h, RT), washed twice with tap water and stained with Oil Red (0.35% in 6: 4 EtOH:water, 15 min at RT followed by two dH_2_O washes). Coverslips were mounted in Crystal-Mount (Electron Microscopy Sciences, Hatfield, PA) for imaging. Live cell experiments comprised 30 min staining at 37°C in media with 4 µM Fluo-4 and 0.05% ORO from 5% stock in 70: 30 water: EtOH.

### Imaging

Bright field and fluorescence imaging of cells in MatTek dishes (50,000 cells per cm^2^) were performed on a Nikon Ti Eclipse C1 epi-fluorescence and confocal microscopy system, equipped with heated stage. Available laser lines in FITC, TxRed and Cy5 were supplied by a 488 nm 10 mW solid state laser, a 561 nm 10 mW diode pump solid state (DPSS) laser and a 638 nm 10 mW modulated diode laser. Z stack sizes ranged from 3–8 microns depending on the cell being imaged. Each z disc (optical section) ranged from 0.15–1 micron. Pinhole size for all images was 60 microns. Images were analysed in NIS Elements (Nikon, Melville, NY). Microapplication was accomplished with Eppendorf CellTram Vario and Femtojet microapplicator systems controlled by an Eppendorf micromanipulator (Eppendorf, Hamburg, Germany), unless otherwise stated images were acquired through a Plan Apo VC 100X 1.40 oil objective (Nikon). Excitation and emission of dyes used are as follows: ORO, Ex 561 nm/Ex 590/50 nm; Fluo-4, Ex 488 nm/Ex 515/30 nm.

### Leukotriene C_4_ assay

RBL2H3 were treated as indicated and stimulated *via* the FcεRI or using PMA/ionomycin. After a 1 h incubation, supernatants were assayed for the concentration of LTC_4_ using a specific EIA kit (Cayman Chemicals, Ann Arbor, MI) and in reference to a standard curve. Color development proceeded for 45 min and absorbance was read at 405 nm.

### Beta-hexoseaminidase assay

RBL2H3 were plated in cluster plates at 5 × 10^4^ cells/well. Monolayers were washed and incubated in 200 µl Tyrode’s buffer before stimulating as described. After 45 min at 37°C, 25 µL supernatant was removed, clarified by microcentrifugation, and transferred to a 96 well plate containing 100 µL per well 1 mM p-N-acetyl glucosamine (Sigma) in 0.05 M citrate buffer pH 4.5. After 1 hour at 37°C reactions were quenched by the addition of 100 µL per well 0.2 M glycine, pH 9.0. Beta-hexosaminidase levels were read as OD at 405 nm. Results are shown as the mean ± standard deviation.

### Flow cytometry

Cells were fixed in 0.4% paraformaldehyde (45 min, RT) and resuspended at 1 × 10^6^ cells/ml in FACS buffer (HBSS, 0.5% BSA, 0.05% NaN_3_). For lipid body analysis, cells were stained with Nile Red (0.1 µg/ml, 30 min RT). For berberine sulfate staining, cells were incubated with 0.025% BS, pH 4.0 (20 min RT) [29]. For immunocytochemistry analysis, cells were harvested as above and resuspended in FACS buffer. Flow cytometry controls were performed to block non-specific binding of primary and secondary antibodies to surface FcεR (Fc gamma receptors) on mast cells. Unpermeabilized cells were blocked for 1 h with BSA followed by rabbit anti-mouse mixed IgGs. Permeabilization was with 0.1% TX100 for 4 min, followed by washing. Primary antibodies were incubated for 30 min at RT at a concentration of 0.1–1 µg/ml. After three washes, cells were incubated with 0.05 µg/ml of the indicated Alexa-coupled secondary antibody (Invitrogen). Fluorescence was assessed on a FACSAria Flow Cytometer (BD Bioscience, Franklin Lakes, NJ) at the John A. Burns School of Medicine Flow Cytometry Facility. Flow cytometry data were analyzed in FlowJo version 9.02.

### Analysis

Analysis of microarray data is described above. For other data types results are shown as the mean+standard deviation. Statistical significance was determined based on a two-way analysis of variance (Student’s t-test). Adjacent to data points in the respective graphs, significant differences were recorded as follows: single asterisk, p<0.05; double asterisk, p<0.01; triple asterisk, p<0.001; no symbol, p>0.05. Experiments are all n of at least 3.

## Results

### Altered SG: LB ratio and associated functional plasticity in chronically insulin-treated model mast cells

**Figure 1:**
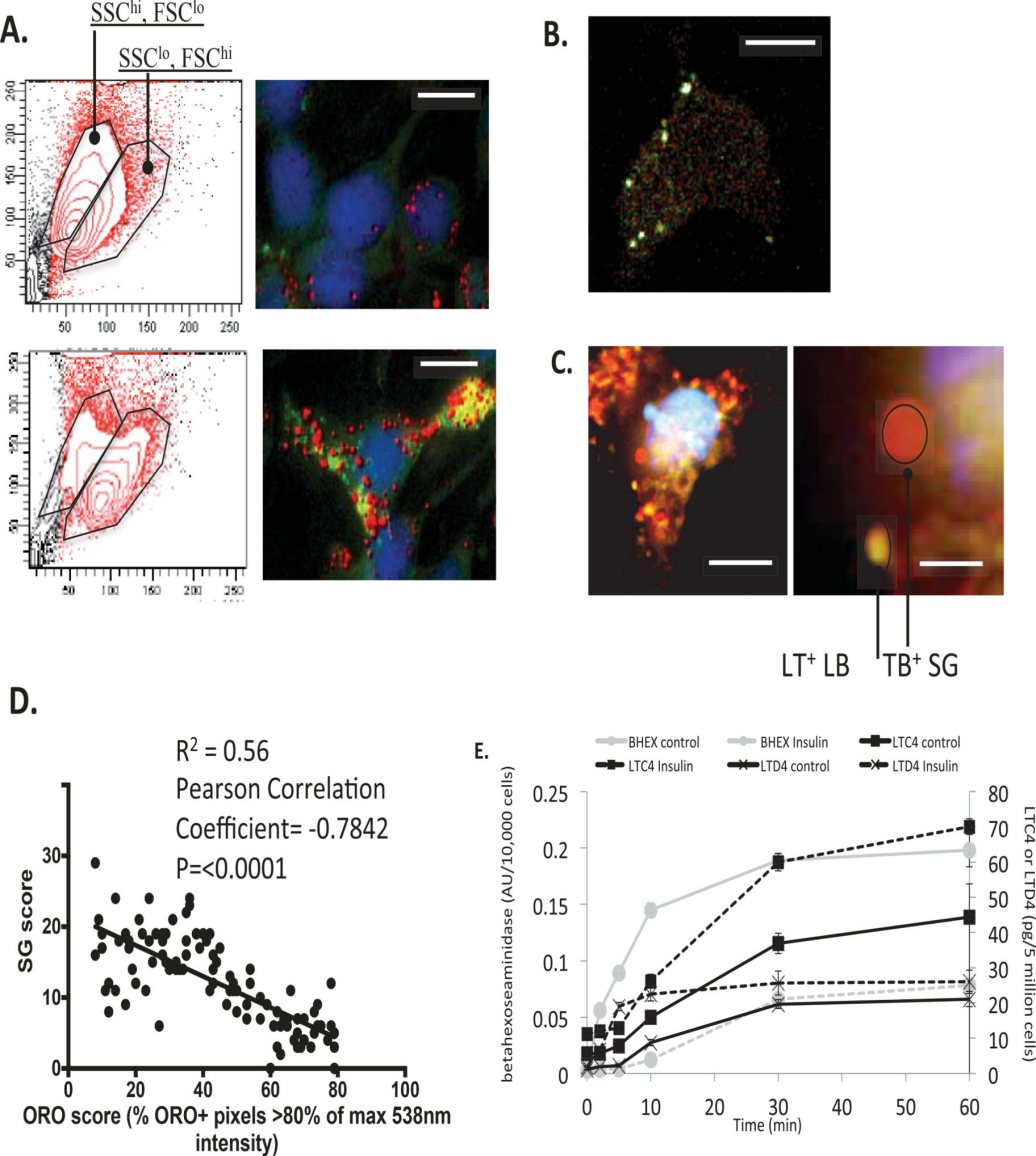
Altered SG: LB ratio and associated functional responses in chronically insulin-treated RBL2H3 **A:** Scatter profile and reference images of control and insulin-FDI-treated MC. Left panels. RBL2H3 cells were incubated with insulin treatment, or left untreated, for 6 days. Cells (5 × 10^5^) per sample were harvested and fixed in PFA before being assessed for cell size (FSC) and granularity/membrane complexity (SSC). Right panels. Fluorescent visualization of lipid bodies using Oil Red O (ORO, 0.35% w/v) counterstained with DAPI in PFA-fixed RBL2H3 cells either untreated or treated with 10 µg/ ml insulin for 6 d. Deconvolved z stacks were projected as an extended depth of focus (EDF) image using Nikon NIS Elements. Scale bars are 5 microns. **B:** Toluidine Blue (TB) fluorescence and anti-tryptase staining co-localize in RBL2H3. PFA-fixed RBL2H3 were stained with TB and anti-tryptase (0.1 µg/ml for 1 h, followed by an Alexa-488 conjugated anti-rabbit IgG) and imaged as described in Methods. False color assignment of red (TB), green (tryptase) and white (colocalization) was assigned in post-processing. **C:** Inverse correlation between secretory granule and ORO levels in RBL2H3. Toluidine Blue and LipiTox Green (LT) were used to fluorescently label SG and LB, respectively. Left panel: Representative cell showing TB staining in red, LT staining in green/gold and DAPI in blue. Scale bar is 10 microns. Right panel: Higher magnification image showing TB staining in red, LT staining in green/gold and DAPI in blue. Scale bar is 1 micron. **D:** Inverse correlation between secretory granule and ORO levels in RBL2H3. Toluidine Blue and LipiTox Green (LT) were used to fluorescently label SG and LB, respectively. Left panel: Representative cell showing TB staining in red, LT staining in green/gold and DAPI in blue. Scale bar is 10 microns. Right panel: Higher magnification image showing TB staining in red, LT staining in green/gold and DAPI in blue. Scale bar is 1 micron. ROI (Regions of Interest) for both were established using a binary overlay and diameters in representative z discs were used to calculate % area and plot represents inverse relationship between SG and LB number in 15 Insulin-treated cells each sliced into 144 z discs. The secretory score (Y) measures the number of SG (they are few in number and clearly delineable) and LB+ area (X, # of ORO* pixels since the LB cluster and are difficult to score as individual organelles). Pearson correlation coefficient for 100 analyzed XY pairs derived from 3 cells was-0.7482 and the 95% confidence intervals for the correlation was −0.86 to −0.64. The p value (two-tailed) for this correlation is <0.0001 (****, significant). **E:** This figure displays beta-hexoseaminidase (BHEX), Leukotriene C_4_ (LTC_4_) and Leukotriene D_4_ (LTD_4_) release from control and insulin treated RBL2H3 cells over 60 min. The left vertical axis displays BHEX release in units/10,000 cells while the right vertical axis displays LTC_4_ and LTD_4_ release in pg/5 million cells.

In prior work we have identified that chronic hyperinsulinemia, applied either *in vitro* or *in vivo*, causes mast cells to assume a steatotic state, loaded with complex lipid bodies (LB). This loading of the cytoplasm with LB (Figure 1A, left panel) is associated with an overall loss in granularity (Figure 1A, right panel). Flow cytometric analysis reveals that chronic hyperinsulinemia results in a population shift of mast cells in terms of scatter profile; a slight in increase in forward scatter (FSC) and a marked decrease in side scatter (SSC) occur (Table 1). SSC is a surrogate measurement for both surface complexity and cytoplasmic granularity and on this basis we hypothesized that lower secretory granule (SG) numbers may be present in the insulin-treated cells. We tested this both histologically and functionally. Insulin-treated cells displayed an approximately 66% reduction in the number of Toluidine Blue-positive structures per cell compared to controls (p=0.002, data not shown). Toluidine blue (here examined fluorescently) co-localizes with anti-tryptase immunofluorescence (Figure 1B). A combination of Lipidtox and TB staining can be used to discriminate LB and SG in the same cell (Figure 1C, right panel). The number of SG and LB are negatively correlated following Insulin treatment (Figure 1D and Table 1). Functionally, we noted a relative suppression in FcεRI-induced beta-hexoaseaminidase release (an assay for secretory granule capacity) and a concomitant relative increase in FcεRI-induced leukotriene C_4_ or D_4_ release (Figure 1E). LTC_4_ is partially synthesized from LB-stored precursors in the arachidonic acid pathway, and thus its elevation may represent an increase in the available pool of LTC_4_ precursors for de novo synthesis of the eicosanoid mediator present in the elevated numbers of LB seen in insulin-exposed cells.

### Overview of transcriptional reprogramming in chronically insulin-treated model mast cells

**Figure 2:**
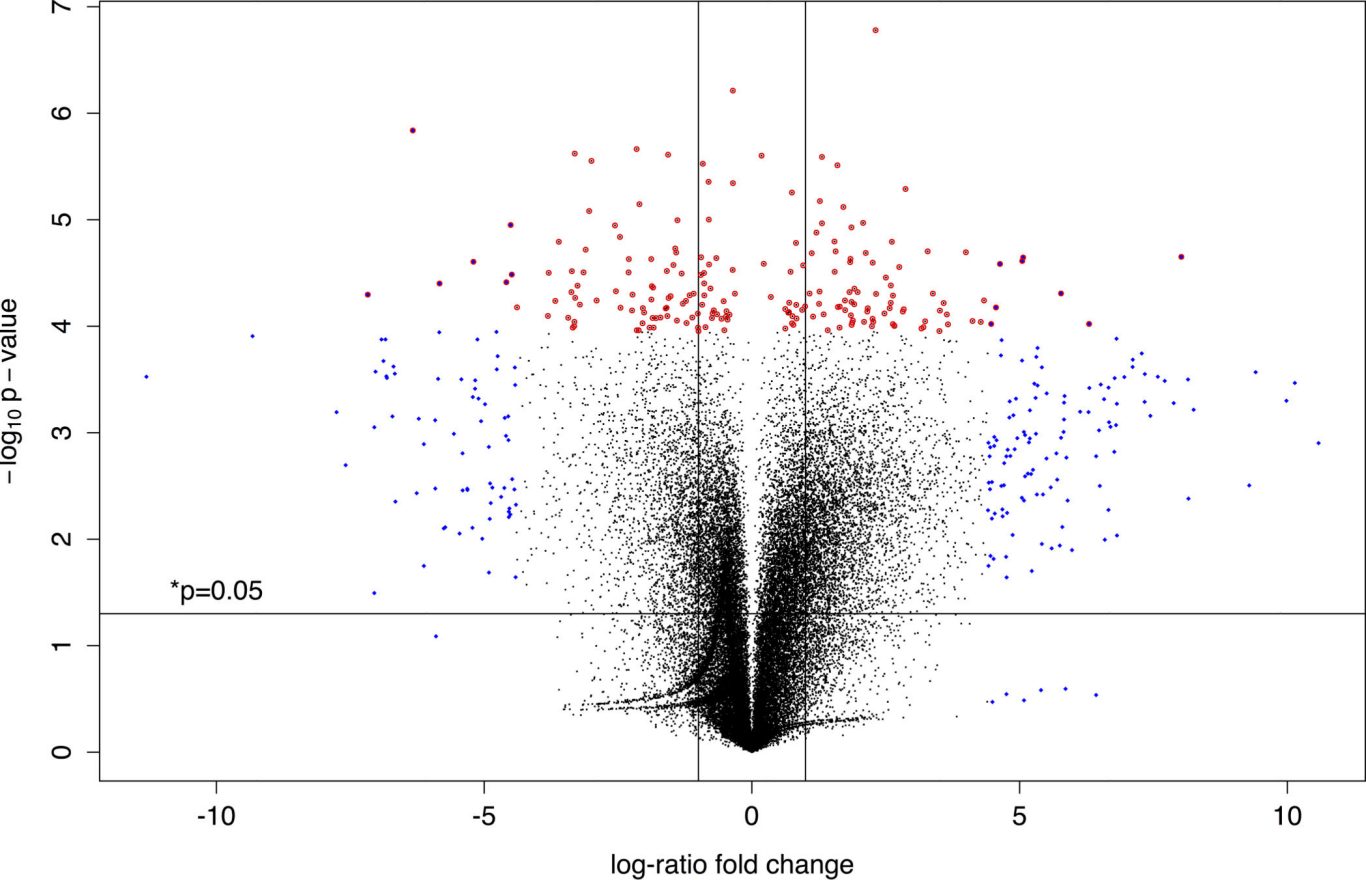

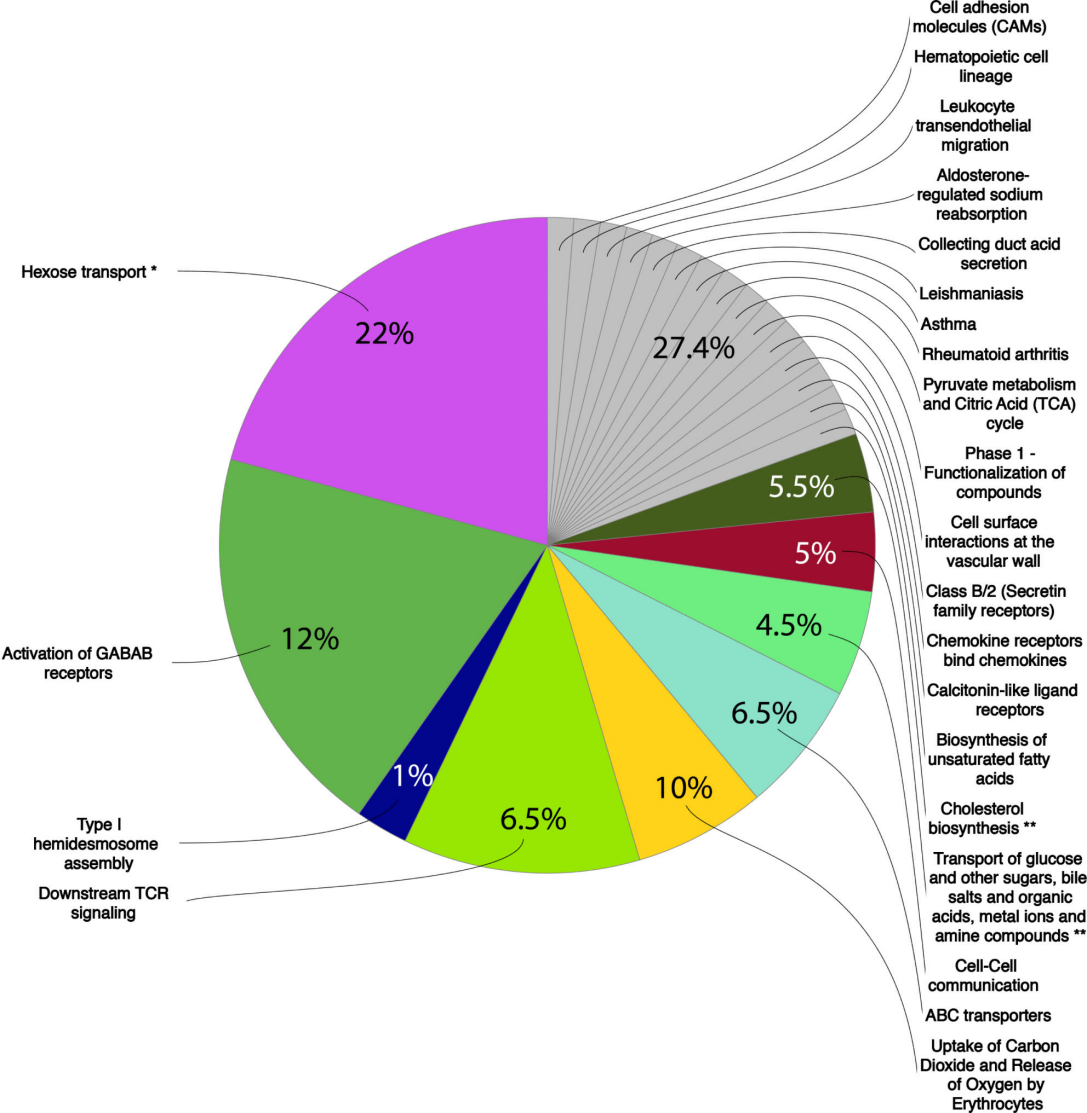

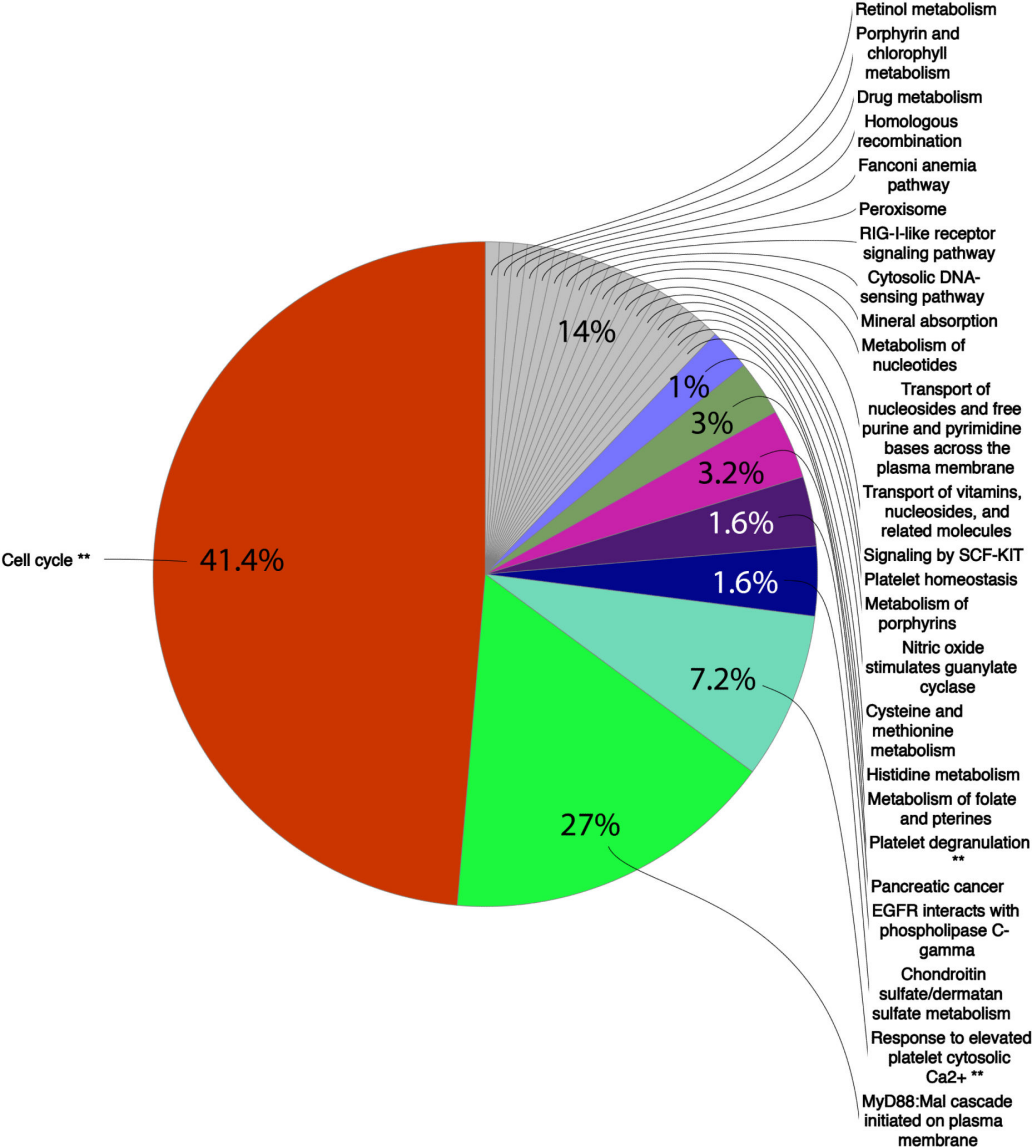
**A:** Overview of transcriptional reprogramming in chronically-insulin treated cells. This volcano plot visualizes the differential expression of all the gene probes on an Agilent Technologies Whole Rat Genome Microarray, which displays 43,379 biological features. The figure plots the x-axis as the log_10_ fold change of the treatment effect on represented genes versus the t-test -log_10_ p-values of the same genes. Lines and letters have been added as guides to help distinguish regions within the plotting area. A single horizontal line intersecting 3 on the y-axis delineates a p-value of 0.001 or less above and greater below the line. Two vertical lines intersecting at −2.5 and 2.5 on the x-axis delineate a log_10_ fold change <-2.5 or >2.5 to the left or the right of those two lines, respectively. The top 200 probes with log_10_ fold changes <-2.5 or >2.5 are plotted with blue diamonds. The top 200 probes with p-values <0.001 are plotted with red open circles. Probes whose values intersect both criteria are red circles with blue centers. Regions: A (down regulation and low p-value), B (up regulation and low p-value), C (down regulation), D (up regulation), E (low p-value), where used to help select candidate genes for further investigation. Probe labels were added to the highlighted genes above the 0.001 p-value line to aid in discovery. **B:** Gene Ontology (GO) Enrichment Tables-down regulation. Pie chart with analysis of the top two hundred biological process ontology terms for genes with the lowest p-value score that were down regulated. The chart is grouped into 24 different processes and represents the ontologies of 609 genes. **C:** GO Enrichment Tables-up regulation. Pie chart with analysis of the top two hundred biological process ontology terms for genes with the lowest p-value score that were up regulated. The chart is grouped into 26 different processes and represents the ontologies of 201 genes.

We performed a transcriptional array analysis to assess the changes in gene expression that are associated with the phenotypic shift that chronic insulin exposure engenders in model mast cells. We initially visualized the whole altered transcriptome using a volcano plot (Figure 2A) which charts gene regulation in terms of fold changes against the statistical significance level of that change. This visualization allows us to achieve resolution that is especially helpful in the cases of genes with small absolute fold expression changes (FEC), but high statistical significance (SS), which can be highly biologically relevant even though the fold change is low. We categorised genes into 5 classifications: A, FEC ≤ −1.5, SS ≥ 1.3 (p=0.05); B, FEC ≥ 1.5, SS ≥ 1.3; C, FEC ≤ −1.5, SS < 1.3; D, FEC ≥ 1.5, SS <1.3; E, −1.5< FEC < 1.5, SS ≥ 1.3. The numbers of genes appearing in each of these categories are 729 (A), 2523 (B), 470 (C), 418, (D) and 3538 (E), respectively. Immediately obvious enrichments in certain areas of the Volcano plot were noted, and included the presence of secretory granule cargo and biosynthesis genes in area A, with lipid body biosynthesis genes in areas E and B. These gene classes were analysed further (see below).

We performed a Gene Ontology (GO) enrichment analysis on this transcriptional data set. The top 200 genes with altered transcription are presented in Figures 2B and 2C We noted trends in Figure 2B that highlighted hexose transport; activation of GABA B receptors; uptake of carbon dioxide and release of oxygen by erythrocytes; downstream TCR signalling; ABC transporters; cholesterol biosynthesis; transport of glucose and other sugars bile salts and organic acids, metal ions and amine compounds; cell-cell communication and type I hemidesmosome assembly showed the most altered transcription. Figure 2C showed high levels of regulation in the following GO categories: cell cycle; MyD88: Mal initiated on plasma membrane; response to elevated platelet cytosolic Ca^2+^; pancreatic cancer; platelet degranulation; EGFR interacts with phospholipase C gamma; chondroitin sulfate/dermatan sulfate metabolism and metabolism of folate and pterines.

### Lipid body biogenesis and lipid metabolism gene sets are regulated in chronically insulin treated model mast cells

**Figure 3:**
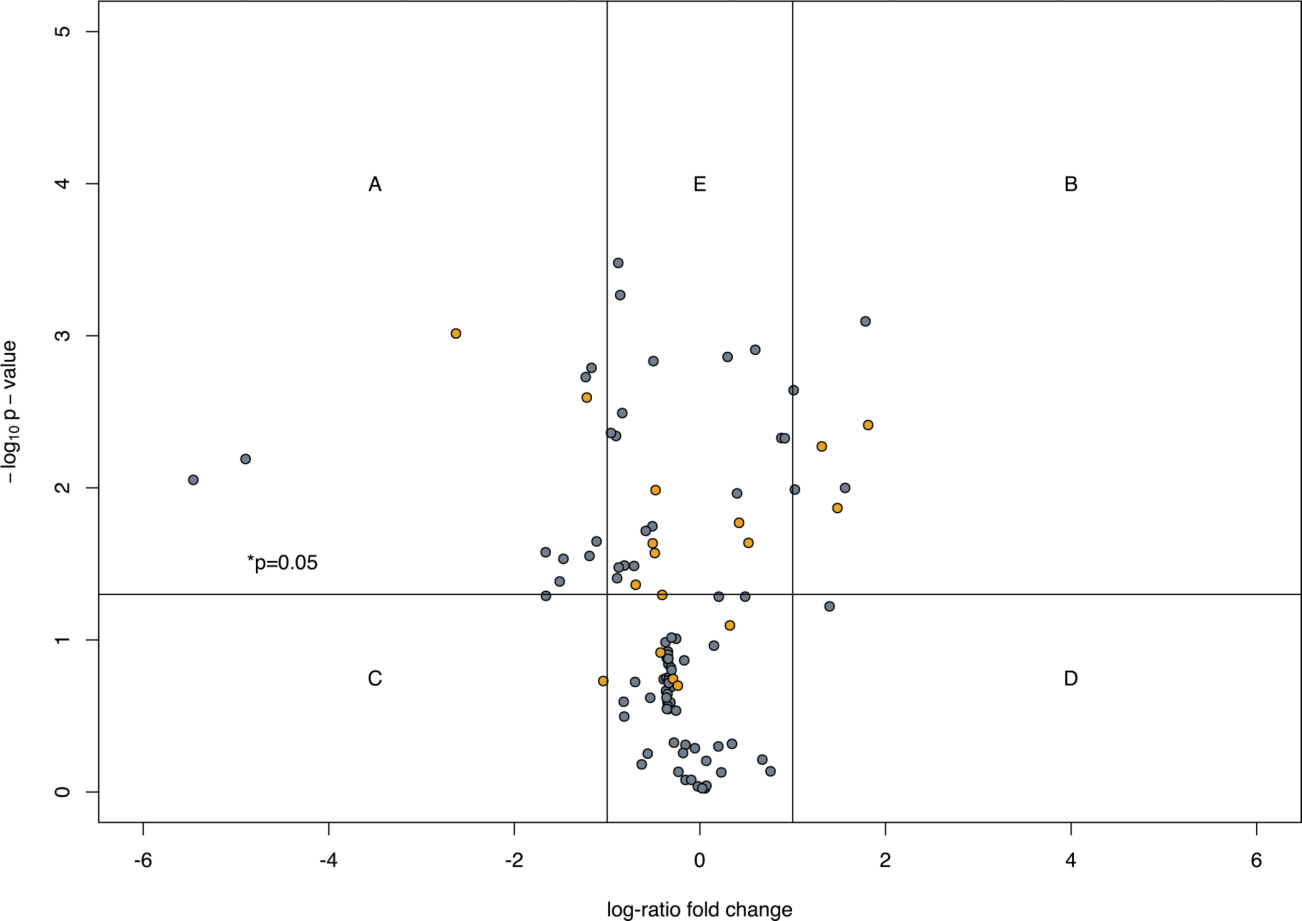

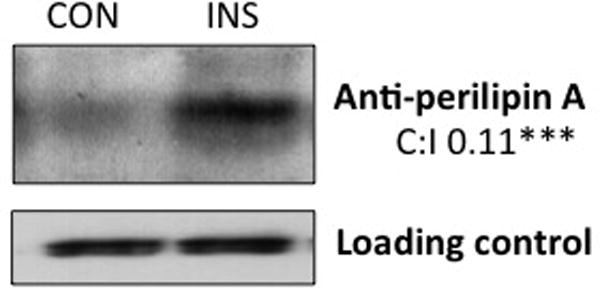

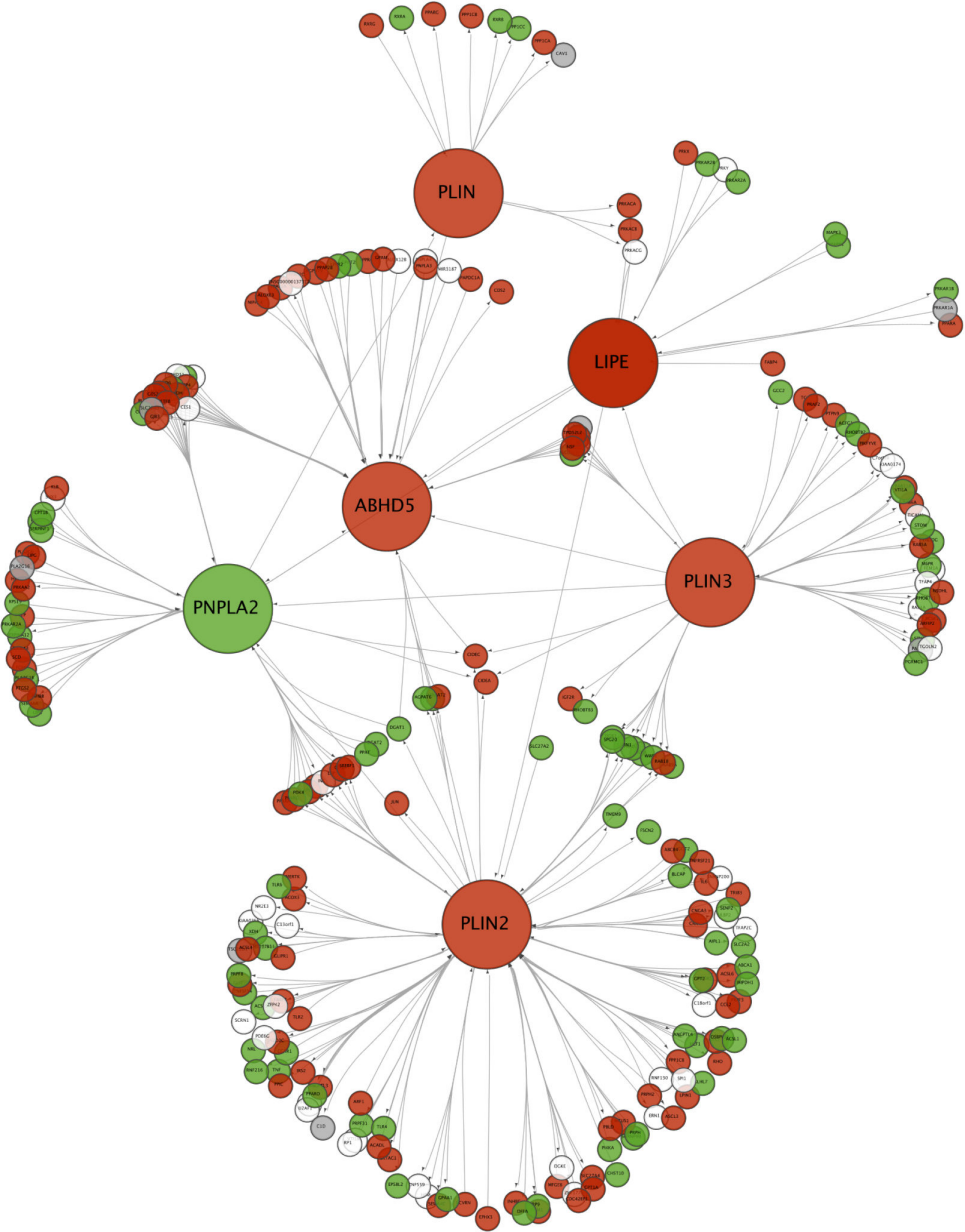
**A:** Lipid biosynthesis genes. Lipid metabolism genes: This volcano plot visualizes the differential expression of all the lipid metabolism gene probes on an Agilent Technologies Whole Rat Genome Microarray, which displays 43,379 biological features. The figure plots the x-axis as the log_10_ fold change of the treatment effect on represented genes versus the t-test -log_10_ p-values of the same genes. Lines and letters have been added as guides to help distinguish regions within the plotting area. A single horizontal line intersecting 2 on the y-axis delineates a p-value of 0.01 or less above and greater below the line. Two vertical lines intersecting at −2.0 and 2.0 on the x-axis delineate a log_10_ fold change <-2.5 or >2.5 to the left or the right of those two lines, respectively. The top 50 probes with log_10_ fold changes <-2.0 or >2.0 are plotted with blue circles. The top 50 probes with p-values <0.01 are plotted with orange open circles. Probes whose values intersect both criteria are red circles with blue centers. Regions: A (down regulation and low p-value), B (up regulation and low p-value), C (down regulation), D (up regulation), E (low p-value), where used to help select candidate genes for further investigation. Probe labels were added to the highlighted genes above the 0.01 p-value line to aid in discovery. **B:** Lipid biosynthesis genes. Example validation of low p-value gene upregulation: Anti-Perilipin A Western blots were performed on RBL2H3 stimulated for 6 days with vehicle (CON) or 10 µg/ml insulin (INS). Quantification of Western blot signal using ImageJ is shown at left. Western blot for the unrelated signalling protein Grb2 (lower panel) was used as a loading control. **C:** Lipid biosynthesis genes. Lipid body biogenesis genes: The expression of essential lipid body biosynthetic genes and the protein interactions of 1^st^ neighbors in insulin-treated RBL2H3 cells. Six key genes were selected for examination and compared for relative expression changes with known 1^st^ neighbor interactions of their protein products: Abhd5, 1-acylglycerol-3-phophate O-acyltransferase ABHD5; Lipe, hormone-sensitive lipase; Plin, perilipin-1; Plin2, perilipin-2; Plin3, perilipin-3; Pnpla2, patatin-like phospholipase domain-containing protein 2. The protein interaction network (PIN) graph color-coding is based on the gene expression comparison of IFDI treated cells versus non-treated cells. Red indicates lower expression, green indicates increased expression, gray indicates an ambiguous response across multiple probes, and white indicates insufficient data.

We evaluated the transcriptional array data set for classes of genes involved in Lipid Particle Organization (GO: 0034389) (Figures 3A and 3B). Here, we compare cells that are untreated versus those at the end of 6 d insulin exposure. Since LB accumulation starts at day 2 and is essentially complete by day 4, as expected the LB biosynthetic pathways are not strongly upregulated at the day 6 timepoint. However, certain LB biosynthetic genes remain upregulated (*Akap1*, *Faf2*, *Katna1*, *Prpf19* and *Pnpla2*), as do genes that are involved in forming the very large numbers of phosphatidylcholine (PC) molecules form the unilamellar coat of each LB. We noted significant transcriptional upregulation (p<0.01) of PC synthesizing enzymes *Pcyt1a&b* and pemt (Choline-phosphate cytidylyltransferase A and B and Phosphatidylethanolamine N-methyltransferase, respectively).

At the protein level, a timecourse of expression shows that the expression of an early gene in the LB biosynthesis pathway (TIP47) peaks around day 3 of insulin exposure (not shown), while Perilipin levels are sustained more chronically (Figure 3B) despite being transcriptionally downturned by day 6 (as shown by the force-directed network analysis in Figure 3C).

### The increased LB to SG ratio in chronically insulin-treated model mast cells is associated with decreased transcription of SG biogenesis and cargo genes

**Figure 4:**
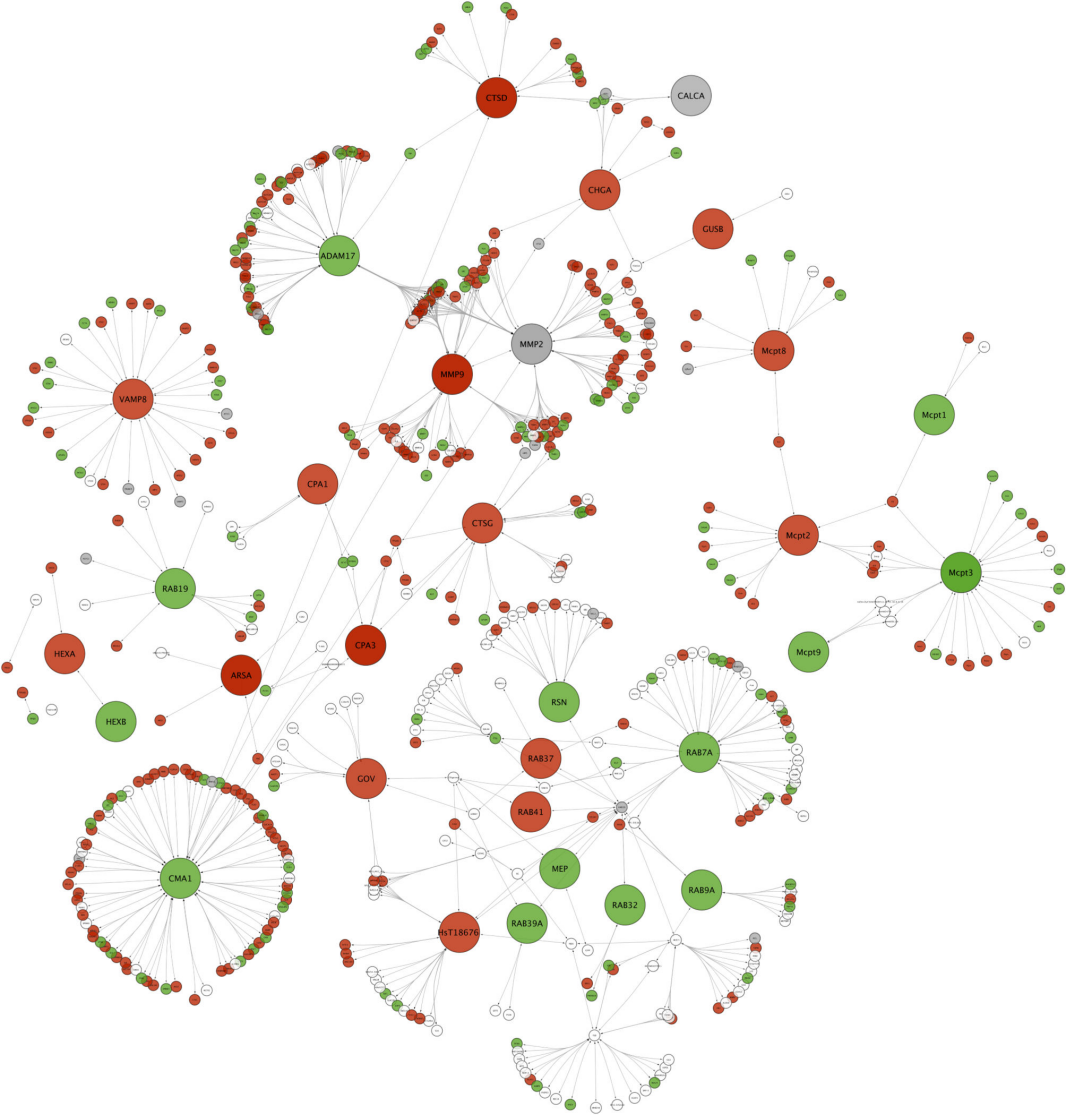

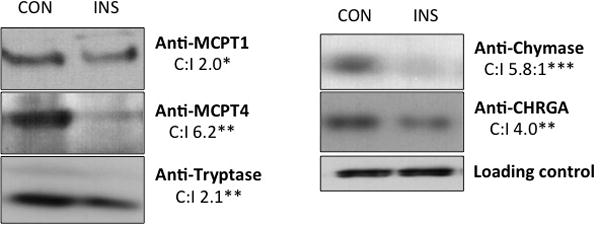

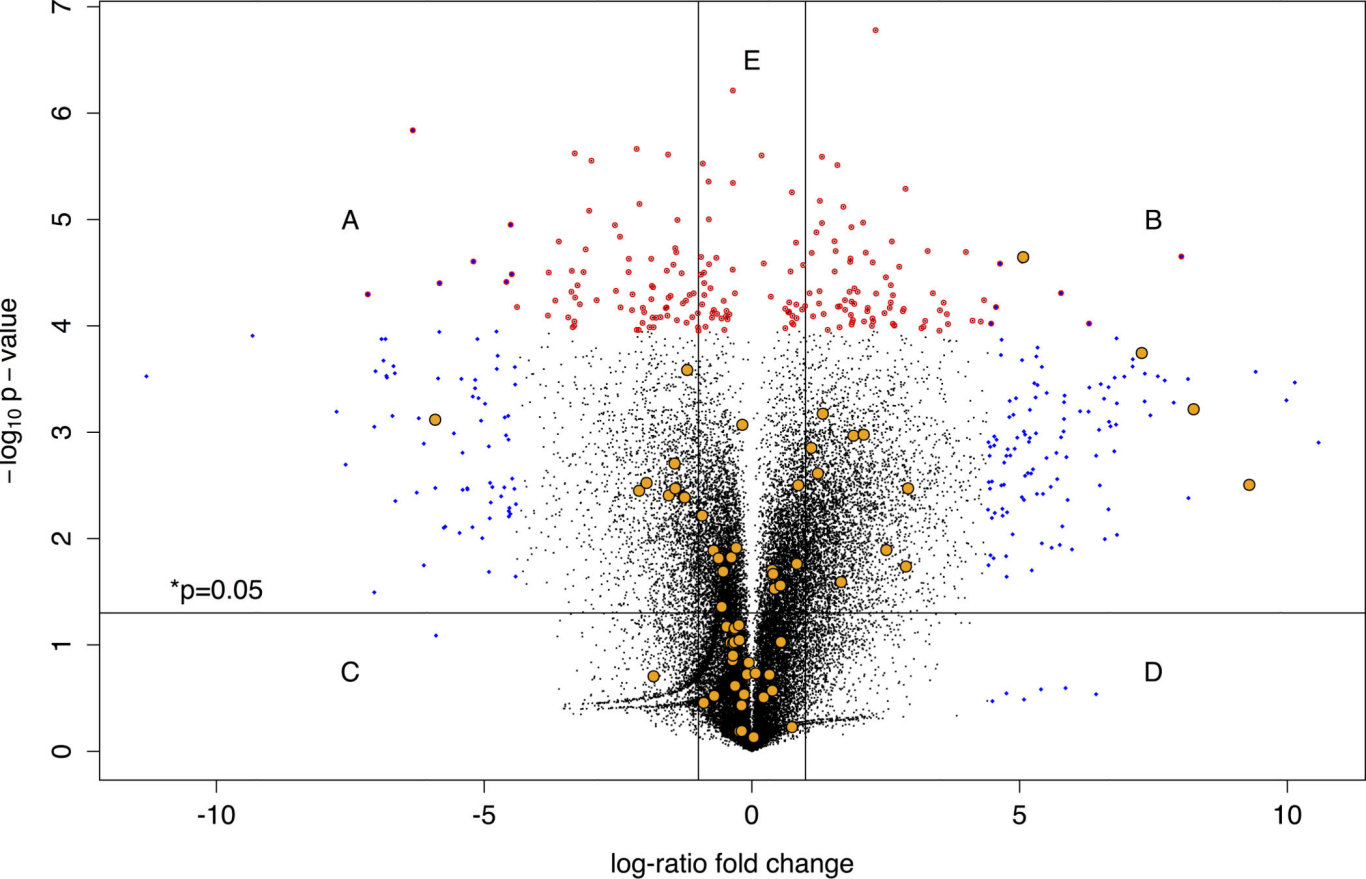
**A:** Secretory granule biogenesis and cargo genes. Secretory granule biogenesis and cargo genes. The expression of essential secretory granule biogenesis or cargo genes and the protein interactions of 1^st^ neighbors in insulin treated versus control RBL2H3 were examined. Thirty-one key genes were selected for examination and compared for relative expression changes with known 1^st^ neighbor interactions of their protein products: Adam17, disintegrin and metalloproteinase domain-containing protein 17; Arsα, arylsulfatase alpha; Calca, calcitonin gene-related peptide 1; Chgα, chromgranin-alpha; Cma1 (Mcpt3), chymase; Cpα1, carboxypeptidase alpha1; Cpα3, mast cell carboxypeptidase alpha; Ctsδ, cathepsin delta; Ctsγ, cathepsin gamma; Gov (Rab3δ), RAS-related protein-3delta; Gusβ, beta-glucuronidase; Hexα, beta-hexosaminidase subunit alpha; Hexβ, beta-hexosaminidase subunit beta; Hst18676 (Rab27α), ras-related protein RAB-27alpha; Mep (Mep1α), meprin a subunit alpha; Mmp2, 72 kDa type IV collagenase; Mmp9, matrix metalloproteinase-9; Mcpt1, mast cell protease 1; Mcpt2, mast cell protease 2; Mcpt3 (Cma1), chymase; Mcpt8, , mast cell protease 8; Mcpt9, granzyme J; Rab19, RAS-related protein RAB-19; Rab32, RAS-related protein RAB-32; Rab37, RAS-related protein RAB-37; Rab39A, RAS-related protein RAB-39A ; Rab41, RAS-related protein RAB-41; Rab7A, RAS-related protein RAB7A; Rab9A, RAS-related protein RAB-9A; Rsn(Clip1), CAP-gly domain-containing linker protein 1; Vamp8, vesicle-associated membrane protein 8. The protein interaction network (PIN) graph color-coding is based on the gene expression comparison of T1DM DRlyp/lyp rat derived mast cells versus DR+/+ rat derived mast cells. Red indicates lower expression, green indicates increased expression, gray indicates an ambiguous response across multiple probes, and white indicates insufficient data. **B:** Secretory granule biogenesis and cargo genes. Example validation of gene upregulation. Anti-Mast cell Protease 1 (MCPT1), Mast Cell Protease 4 (MCPT4), Tryptase, Chymase or Chromogranin A (CHRGA) Western blots were performed on RBL2H3 stimulated for 6 days with vehicle (CON) or 10 µg/ml insulin (INS). Quantification of Western blot signal using ImageJ is shown at left. Western blot for the unrelated signalling protein Grb2 (lower panel) was used as a loading control. **C:** Secretory granule biogenesis and cargo genes. Volcano Plot. This volcano plot visualizes the differential expression of all the gene probes on an Agilent Technologies Whole Rat Genome Microarray, which displays 43,379 biological features. The figure plots the x-axis as the log_2_ fold change of the treatment effect on represented genes versus the t-test -log_10_ p-values of the same genes. Lines and letters have been added as guides to help distinguish regions within the plotting area. A single horizontal line intersecting 3 on the y-axis delineates a p-value of 0.001 or less above and greater below the line. Two vertical lines intersecting at −2.5 and 2.5 on the x-axis delineate a log_2_ fold change <-2.5 or >2.5 to the left or the right of those two lines, respectively. The top 200 probes with log_2_ fold changes <-2.5 or >2.5 are plotted with blue diamonds. The top 200 probes with p-values <0.001 are plotted with red open circles. Probes whose values intersect both criteria are red circles with blue centers. Essential secretory genes are highlighted as orange circles. Regions: A (down regulation and low p-value), B (up regulation and low p-value), C (down regulation), D (up regulation), E (low p-value), where used to help select candidate genes for further investigation. Probe labels were added to the highlighted genes above the 0.001 p-value line to aid in discovery.

We mined the transcriptional array and performed confirmatory Western blots in order to assess the expression levels of genes involved in either the biogenesis of SG or that constitute cargo for SG. These sets are somewhat overlapping, since prior studies in other cell systems (neuroendocrine cells and pancreatic beta cells) suggest that the presence of cargo itself drives SG biogenesis [30]. Cargo genes (mast cell proteases), the granin family (cargo and SG biogenesis genes) and genes such as the Rab family members that are involved in SG trafficking and maturation were examined. The force-directed network analysis in Figure 4A shows that genes such as mast cell proteases (mcpt2, mcpt8, carboxypeptidases, MMPs) as well as Rab proteins involved in secretory granule formation, plasma membrane docking and exocytosis are downregulated. At the protein level, the cells post 6 d insulin displayed diminished levels of several mast cell proteases (Figure 4B). These gene expression changes support the observation of reduced SG numbers that we observe (Figure 1B) and the accompanying diminished intensity of beta-hexoseaminidase release in insulin-exposed cells.

Within the set of negatively-regulated genes in chronically insulin-treated cells we sought patterns that could reveal gene sets that are targets for the suppression of SG formation, a potential anti-allergy strategy. Figure 4C shows a projection of GO: 0032254 (establishment of secretory granule localization), GO: 0032255 (maintenance of secretory granule location), GO: 0030141 (secretory granule), GO: 0033363 (secretory granule organization), GO: 0033364 (mast cell secretory granule organization), GO: 0033367 (protein localization to mast cell secretory granule), GO: 0033368 (protease localization to mast cell secretory granule), and GO: 0061109 (dense core granule organization) onto the negatively regulated genes identified in Figure 2A.

### Glucagon and TNF alpha reversal of insulin-induced plasticity in mast cell phenotype

In adipocytes and hepatocytes insulin and nutrient excess drive lipid droplet accumulation and steatosis. This is a reversible process, with glucagon and TNF alpha identified as stimuli that can drive a decrease in lipid droplet load and decrease expression of inflammatory and other steatotic markers [31–33]. We examined the potential for this reversal in insulin-treated mast cells. First, we validated the effects of glucagon and TNF alpha exposure after insulin withdrawal in the 3T3 adipocyte cell line and in primary adipocytes, where glucagon reversed lipid body accumulation by ~80% by 6 days exposure and TNF alpha achieved a maximal reversal of 22% at 9 days (data not shown). We also initially compared microscopic and spectrophotometric methods for measurement of ORO staining, found them to be broadly comparable (not shown) and then focused exclusively on the microscopic method.

Table 2 shows a comparison of ORO staining density in RBL2H3 cells in response to a time course and dose response of glucagon and TNF alpha. (i) We performed a time course and dose response of glucagon and TNF alpha exposure in model mast cells previously incubated with insulin for 6 days. LB content was assessed using quantification of ORO-positive area in triplicate z discs from 50 cells per point. Stimuli comprised 6 day exposure to insulin (10 µg/ ml) for 6 days followed by exposure (with matched passaging) to glucagon (10 µg/ml) or TNF alpha (250 nM) (unless concentrations otherwise specified). ORO-positive area at initial timepoint or zero dose was treated as 100%, then effects of stimuli were expressed as % normalized to the starting value. Timecourse data show that by 9 d of exposure, glucagon reduced the percentage of ORO positive z disc area by ~20% and 62%, respectively, both at a significance of p<0.05. Dose response data shows that maximal effects of glucagon and TNF alpha for reduction or reversal of insulin-induced steatosis are active at >1 µg/ml glucagon and 100 nM TNF alpha, respectively. (ii) We compared the percentage reversal of 6 d insulin-induced steatosis at 9 day exposure to vehicle. Glucagon or TNF alpha, showing that for the RBL2H3 TNF alpha is more effective than glucagon (in contrast to 3T3-L1 adipocytes). (iii) We assessed leukotriene C_4_ secretion in response to antigenic stimulation of the RBL2H3 *via* FcεRI in cells exposed to 6 d insulin then 9 d vehicle, glucagon or TNF alpha. LTC_4_ release we have previously demonstrated to be enhanced over control levels by 6 d insulin treatment, but 9 d glucagon or TNF alpha results in a decrease in PTC_4_ secretion in response to matched doses on antigen. Here, again, TNF alpha shows a more substantive effect than glucagon. (iv) TNF alpha is also effective at decreasing the LTC_4_ secretion in non-insulin exposed cells, likely reflecting that decreased LB load reflects a decreased precursor pool for LTC_4_ biosynthesis. (v) Secretory granule numbers do not recover as LB numbers decrease due to reversal of steatosis by TNF alpha. These data show that the ORO-positive cell area in 6 day insulin treated cells subsequently exposed to TNF alpha is approximately half of the area on 6 day insulin then vehicle-exposed cells. In contrast, while 6 d insulin induces a decrease in the number of major tryptase positive structures that do not co-localize with ER or Golgi stains (resting levels are median of 36 structures per cell, range from 5–56 structures), these numbers do not recover with exposure to TNF alpha. (vi) Similarly, neither glucagon nor TNF alpha affect the degranulation responses (betahexoseaminidase release) from RBL2H3 subsequent to insulin exposure.

## Discussion

Chronic hyperinsulinemia is a phase of the development of metabolic syndrome (MS) during which systemic levels of insulin are elevated [34]. In this context, mast cells and other immunocytes would find their tissue milieu to be enriched for insulin chronically, and so the reprogramming of mast cells that we observe *in vitro* and *in vivo* has potential relevance to inflammatory responses in patients with MS [6]. Conversely, there are also clinical situations (e.g., type I diabetes) in which mast cells and other inflammatory cells would exist chronically in an insulin-deficient milieu. Here we modelled chronic hyperinsulinemia in a mast cell line. Hyperinsulinemia appears to promote a SG^lo^LB^hi^ phenotype, while conversely, insulin deficiency would be predicted to engender either no change on the SG: LB ratio or a tendency towards SG^hi^: LB^lo^. These data support and extend our prior findings [6] and resonate with prior findings showing that at an epidemiological level, Type I diabetes incidence is negatively correlated with atopy, and that surgically or chemically pancreatectomized animals are broadly protected from anaphylaxis and refractory to anaphylatoxins. All of these studies suggest some linkage between mast-cell mediated responses and the set point of insulin the body [9,10,35,36].

There are of course caveats to our pilot study, specifically the need to provide evidence of altered SG: LB ratio and accompanying transcriptional reprogramming in human mast cells. Primary murine and human basophils exhibit this alteration under conditions of 6 d exposure to high insulin levels *in vitro*, but the SG: LB phenotype of mast cells or basophils exposed to altered insulin levels *in vivo* has not been studied. Further, InsR deficient mast cells or mast cells from INSR-deficient whole animals have not yet been studied for their LB/SG histology, functional phenotype or responses to allergic or anaphylactic stimuli in summary, our data suggest possible plasticity in MC functional phenotype, which in turn may be regulated by the endocrine system and metabolic status.

Our data raise the interesting question of whether insulin plays a role in regulating mast cell functional phenotype *in vivo*. If insulin is an exogenous stimulus that can alter mast cell phenotype, then we can consider that in hyper and hypo-insulinemic clinical situations, mast cell function would be affected. For example, with hyperinsulinemia pushing mast cells towards the SG^lo^LB^hi^ phenotype, the mast cells would be pushed towards LTC_4_ production, which could manifest in an increased tissue inflammation in areas such as airways. The SG^lo^LB^hi^ phenotype could also lead to some benefits such as reduced mast cell degranulation from the increased levels of IgE in those that suffer from atopy. There are interesting studies in pancreatectomized rodents suggesting that insulin-deficiency modulate the intensity and duration of anaphylactic responses characterized by mast cell degranulation into tissue. This creates a need to examine the insulin-deprivation counterpart to our hyperinsulinemia model system, with the latter a question that would need to addressed *in vivo* and/or with insulin receptor-deficient mast cells *in vitro* or *in vivo*.

The implication that the SG: LB ratio can be regulated exogenously have implications for both mast cell functional plasticity and for the generation of mast cell heterogeneity. In terms of plasticity, the presence of insulin receptors on primary mast cells and basophils does suggest that there is an evolutionary adaptation in these cells to be responsive to insulin [6,37]. Gut mast cells may be an important future study target, because insulin is a neuroendocrine signal that is involved in maintaining blood sugar during metabolism and fluctuates over a daily and hourly timecourse response to eating; we can speculate that gut mast cell functionality could be influenced by insulin across shorter time courses than in chronic hyper- or hypo-insulinemia. The ability of glucagon to affect mast cell lipid body load is a novel observation that may be relevant to diurnal feeding cycles in the gut, but in our study it is somewhat overshadowed by the effect size of TNF alpha. TNF alpha has been shown to be lipolytic in adipocytes and is relevant to the modulation of lipid body numbers in neutrophils and macrophages under conditions of infection [38–41]. Our study suggests plasticity in LB size and abundance, and links this to the intensity of bioactive lipid mediator release. Thus the hormones and cytokines tested here may regulate mast cell-derived responses that are relevant to pathological and beneficial inflammations, and tissue homeostasis.

Mast cell heterogeneity has been described across tissue locations, species (between rodents and humans) and functionally [37,42,43]. Markers of this heterogeneity include functional distinctions in the relative production of lipid mediators and histaminergic capacity, and in the expression of distinct patterns of mast cell proteases. The transctiptional regulation of proteases observed in our transcriptional analysis suggests that insulin may not simply be ‘switching’ secretory function to a lower level, but there may be more subtle changes within the complement of MC proteases that are being expressed, which will in turn alter the activated MC’s impact upon the surrounding tissue. Further work will be needed to determine both the regulatory mechanisms and signalling pathways that connect INSR to these targets, and the functional impact of insulin elevation or suppression on mast cell function across a range of physiological and pathophysiological metabolic, and endocrine, scenarios.

## Figures and Tables

**Table 1:** Summary table of flow cytometry and LB/SG staining quantification in control (CON) and 6 d insulin (INS) treated cells.

	CON	INS	P
SSC^hi^, FSC^lo^	68%	32%	***
SSC^lo^, FSC^hi^	27%	73%	***
ORO^+^cytoplasm	11% ± 1.1	36% ± 5.5	***
LT^+^cytoplasm	8% ± 0.08	17% ± 3.4	***
TS^+^cytoplasm	7% ± 0.04	3% ± 1.6	*
Anti-Tryptase^+^structures (mean number per cell >200 nm diameter)	42 ± 17	9% ± 14	*

**Table 2:** Summary data table of glucagon and TNF alpha effects.

**(i) ORO staining density in 6 d insulin treated cell subsequently exposed to glucagon or TNF alpha** average percentage ORO^+^ cell area, normalized to starting percentage, triplicate z discs from n=50 cells							
**Time Course**				**Dose Response**			
Time (d)	Control	Glucagon	TNF alpha	9 d Glucagon	ORO	9 d TNF alpha	ORO
0	100	100	100	0	100	0	100
1	96	96	89	100 ng/mL	92***	0.1 nM	101
2	98	92**	66	1 µg/mL	81***	1 nM	96
4	101	84*	42	10 µg/mL	74***	10 nM	77**
8	96	80**	38			100 nM	52**
12	100	79***	N.D.			1 µM	41***
p value relative to day 0, * p<0.05; **, p<0.01; ***, p<0.001; no symbol, >0.05.				p value relative to zero dose 0, * p<0.05; **, p< 0.01; ***, p<0.001; no symbol, p>0.05.			
**(ii) Reversal of steatosis** average percentage ORO^+^ cell area, triplicate z discs from n=50 cells ± SD, cells exposed to 6 d insulin then 9 d glucagon or TNF alpha)							
**6 d insulin+9 day vehicle**			**+9 d glucagon**		**+9 d TNF alpha**		
ORO%	% reversal		ORO%	% reversal	ORO%	% reversal	
100	−3		100	−11**	100	−31***	
**(iii) LTC**_4_ **secretion** pg/1million cells, in response to IgE (l µg/ml 16h then 250 ng/ml KLH-DNP ± SD							
**6 d insulin+9 day vehicle**			**+9 d glucagon**		**+9 d TNF alpha**		
220 ± 11.2			198 ± 13.6*		106 ± 21.4***		
**(iv) LTC**_4_ **secretion** pg/1 million cells, in response to IgE (1µg/ml 16 h) then KLH-DNP ± SD				**(v) LB and SG relative abundance in response to TNF alpha treatment** ORO^+^ % cell area and anti-tryptase^+^ structures/cell, mean o n=50 cells ± SD			
KLH-DNP (ng/nl)	vehicle	6 d TNF alpha		control		6 d TNF alpha	
0	13.3	12.4		ORO^+^% cell area			
20	15.6	19.3		15.6		7.7**	
200	204.4	164.7**		anti-tryptase^+^ structures/cell			
2000	309.6	212.5**		16 ± 4		13 ± 9	
**(vi) Degranulation responses** Betahexoseaminidase release as % of PMA-Ionomyin stimulated release (% max)							
**6 d insulin+9 day vehicle**			**+9 d glucagon**		**+9 d TNF alpha**		
66.8%			64.9%		63.7%		

(i) ORO staining density in 6 d insulin treated cells subsequently exposed to glucagon or TNF alpha. LB content was assessed using quantification of ORO-positive area in triplicate z discs from 50 cells per point. Stimuli comprised 6 day exposure to insulin (10 µg/ml) for 6 days followed by 9 day exposure (with matched passaging) to glucagon (10 µg/ml) or TNF alpha (250 nM) (unless concentrations otherwise specified). ORO-positive area at initial timepoint or zero dose was treated as 100%, then effects of stimuli were expressed as % normalized to the starting value. (ii) Comparison of the percentage reversal of 6 d insulin-induced steatosis at 9 day exposure to vehicle, versus glucagon (10 µg/ml) or TNF alpha (250 nM). (iii) Leukotriene C_4_ (LTC_4_) release in response to antigenic stimulation of the RBL2H3 *via* FcεRI (1 µg/ml IgE anti-DNP for 16 h followed by 250 ng/ml KLH-DNP) in cells exposed to 6 d insulin then 9 d vehicle, glucagon or TNF alpha. (iv) Leukotriene C_4_ (LTC_4_) release in response to a dose response of antigenic stimulation of the RBL2H3 *via* FcεRI in cells treated with no insulin, but 6 d vehicle or TNF alpha as indicated. (v) LB and SG abundance measured in cells treated with 6 d insulin or 6 d TNF alpha by ORO-positive z disc area (LB) and major anti-tryptase positive structures of >180 nM diameter and not co-localized either ER-or Golgi-specific stains (SG). (vi) Betahexoseaminidase release in response to PMA/Ionomycin expressed as percentage of maximal release in cells exposed to 6 d insulin followed by 9 d vehicle, 9 d glucagon or 9 d TNF alpha.
